# Applying Data-driven Imaging Biomarker in Mammography for Breast Cancer Screening: Preliminary Study

**DOI:** 10.1038/s41598-018-21215-1

**Published:** 2018-02-09

**Authors:** Eun-Kyung Kim, Hyo-Eun Kim, Kyunghwa Han, Bong Joo Kang, Yu-Mee Sohn, Ok Hee Woo, Chan Wha Lee

**Affiliations:** 1 Department of Radiology, Research Institute of Radiological Science and Center for Clinical Image Data Science, Severance Hospital, Yonsei University, Seoul, Korea; 2 Lunit Inc, Seoul, Korea; 3 Department of Radiology, Seoul St. Mary’s Hospital, College of Medicine, The Catholic University of Korea, Seoul, Korea; 4 Department of Radiology, Kyung Hee University Hospital, College of Medicine, Kyung Hee University, Seoul, Korea; 5 Department of Radiology, Korea University Guro Hospital, Seoul, Korea; 6 Department of Radiology, Center for Diagnostic Oncology, National Cancer Center Hospital, National Cancer Center, Gyeonggi, Korea

## Abstract

We assessed the feasibility of a data-driven imaging biomarker based on weakly supervised learning (DIB; an imaging biomarker derived from large-scale medical image data with deep learning technology) in mammography (DIB-MG). A total of 29,107 digital mammograms from five institutions (4,339 cancer cases and 24,768 normal cases) were included. After matching patients’ age, breast density, and equipment, 1,238 and 1,238 cases were chosen as validation and test sets, respectively, and the remainder were used for training. The core algorithm of DIB-MG is a deep convolutional neural network; a deep learning algorithm specialized for images. Each sample (case) is an exam composed of 4-view images (RCC, RMLO, LCC, and LMLO). For each case in a training set, the cancer probability inferred from DIB-MG is compared with the per-case ground-truth label. Then the model parameters in DIB-MG are updated based on the error between the prediction and the ground-truth. At the operating point (threshold) of 0.5, sensitivity was 75.6% and 76.1% when specificity was 90.2% and 88.5%, and AUC was 0.903 and 0.906 for the validation and test sets, respectively. This research showed the potential of DIB-MG as a screening tool for breast cancer.

## Introduction

Mammography is widely recommended for breast cancer screening, although the starting age and screening interval for its application have been debated^1–5^. Screening mammography is recommended as it has a sensitivity over 85% and a specificity over 90%^6^; however, performance varies according to the radiologists’ experience or working area (academic vs nonacademic, general vs specific)^7–9^. Computer-aided detection (CAD) acts as an automated second reader by marking potentially suspicious spots for radiologists to review and several early reports emphasized that this could improve mammographic sensitivity^10–13^, with 74% of all screening mammograms in the Medicare population being interpreted with CAD by 2008^14,15^.

Since the wide introduction of CAD into clinics, radiologists using this technology have complained of a high number of false-positive markers and several recent studies reported that CAD does not improve the diagnostic accuracy of mammography^16,17^. This was somewhat expected. Most learning algorithms including CAD are based on pre-defined hand-crafted features, so they are task-specific, a-priori knowledge based, which causes a large bias towards how humans think the task is performed^18^. Whereas in new algorithms including deep learning, the research has shifted from rule-based, problem specific solutions to increasingly generic, problem agnostic methods^19–21^. This is possible due to the backup of big data, increased computing power and sophisticated algorithms.

The algorithm developed in this study was named data-driven imaging biomarker (DIB; an imaging biomarker derived from large-scale medical image data by using deep learning technology) in mammography (DIB-MG). The basic learning strategy of DIB-MG is weakly supervised learning. Unlike the conventional CAD designs, DIB-MG learns radiologic features from large scale images without any human annotations. So, the purpose of our study was to assess the feasibility of DIB in mammography (DIB-MG) and to evaluate its potential for the detection of breast cancer.

## Materials and Methods

### Data collection

Five institutions (all tertiary referral centers) formed a consortium for the imaging database. All study protocols were approved by the institutional review board of Yonsei University Health System (approval number: 1-2016-0001) and the requirement for informed consent was waived. All experiments were conducted in accordance with the Good Clinical Practice guidelines. For algorithm development, digital mammography images were retrospectively obtained from PACS. We included women with four views of digital mammograms. Exclusion criteria were as follows. 1) Women with previous surgery for breast cancer, 2) Women with previous surgery for benign breast disease within 2 years, 3) Women with mammoplastic bag, 4) Women with mammographic clip or marker. All cancer cases were confirmed by pathology and all normal cases were defined as BI-RADS category 1 (negative) without malignancy development during at least 2 years of follow-up. Both screening and diagnostic mammograms were included. This study was solely focused on whether our algorithm could discriminate cancer from normal cases, so presumed benign cases (BI-RADS categories 2, 3, 4, and 5 without cancer) were not included. Accordingly, 29,107 digital mammogram sets were obtained, in which there were 4,339 cancer cases and 24,768 normal cases. All images in the data sets were recorded by radiologists for breast density, cancer type (invasive vs noninvasive), features (mass, mass with microcalcifications, asymmetry or focal asymmetry, distortion, microcalcification only, etc.) and size of the invasive cancer. For cancers showing mass with microcalcifications, both mass and microcalcifications were recorded as features. Breast density was recorded using BI-RADS standard terminology of almost entire fat (A), scattered fibroglandular densities (B), heterogeneous dense (C), and extremely dense (D)^6^.

### Data sets

In 4,339 cancer cases, training, validation and test sets were randomly selected with a ratio of 5:1:1 (3,101/619/619). Each dataset was evenly distributed in terms of patients’ age, breast density, and manufacturer, and cancer type, feature, and size in order to remove selection bias between training, validation and test sets (Table 1). Predominant features of cancer were mass (n = 2,366) or microcalcifications (n = 1,962), so other features (asymmetry for focal asymmetry (n = 463), distortion (n = 100)) were not controlled in the data sets.

**Table 1:** Demographics in cancer cases.

	Train (n = 3101)	Validation (n = 619)	Test (n = 619)	P value
Density				0.7843
almost entire fat	196 (6.32)	32 (5.2)	31 (5.0)	
scattered fibroglandular densities	640 (20.6)	136 (22.0)	137 (22.1)	
heterogeneous dense	1555 (50.2)	312 (50.4)	312 (50.4)	
extremely dense	710 (22.9)	139 (22.4)	139 (22.5)	
Age				0.9941
≥50	1759 (56.7)	350 (56.5)	350 (56.5)	
<50	1342 (43.3)	269 (43.5)	269 (43.5)	
Manufacturer				0.9351
GE	1226 (39.5)	238 (38.5)	251 (40.6)	
Hologic	1032 (33.2)	200 (32.3)	198 (32.0)	
Siemens	843 (27.2)	181 (29.2)	170 (27.4)	
Feature				
mass	1688 (54.4)	339 (54.8)	339 (54.8)	0.9806
non mass	1413 (45.6)	280 (45.2)	280 (45.2)	
calcifications	1402 (45.2)	280 (45.2)	280 (45.2)	0.9999
non calcifications	1699 (54.8)	339 (54.8)	339 (54.8)	
Type				0.2767
Invasive	2673 (86.2)	542 (87.56)	547 (88.37)	
Noninvasive	428 (13.8)	77 (12.44)	72 (11.63)	
Size (invasive)				0.8409
Size ≥20	1216 (45.5)	254 (46.9)	251 (45.9)	
Size <20	1457 (54.5)	288 (53.1)	296 (54.1)	

In 24,768 normal cases, the same number of validation (n = 619) and test (n = 619) cases were randomly selected, and the rest were used for training. For normal cases, each partition of the dataset was evenly distributed in terms of patients’ age, breast density, and manufacturer in order to remove selection bias (Table 2).

**Table 2:** Demographics in normal cases.

	Train (n = 23530)	Validation (n = 619)	Test (n = 619)	P value
Density				0.898
almost entire fat	837 (3.6)	27 (4.4)	18 (2.9)	
scattered fibroglandular densities	4206 (17.9)	106 (17.1)	115 (18.6)	
heterogeneous dense	16434 (69.8)	432 (69.8)	432 (69.8)	
extremely dense	2053 (8.7)	54 (8.7)	54 (8.7)	
Age				0.997
≥50	14533 (61.8)	383 (61.9)	383 (61.9)	
<50	8997 (38.2)	236 (38.1)	236 (38.1)	
Manufacturer				0.4872
GE	11526 (49.0)	284 (45.9)	315 (50.9)	
Hologic	10191 (43.3)	282 (45.6)	257 (41.5)	
Siemens	1813 (7.7)	53 (8.6)	47 (7.6)	

### Development of the Algorithm

**Figure 1:**
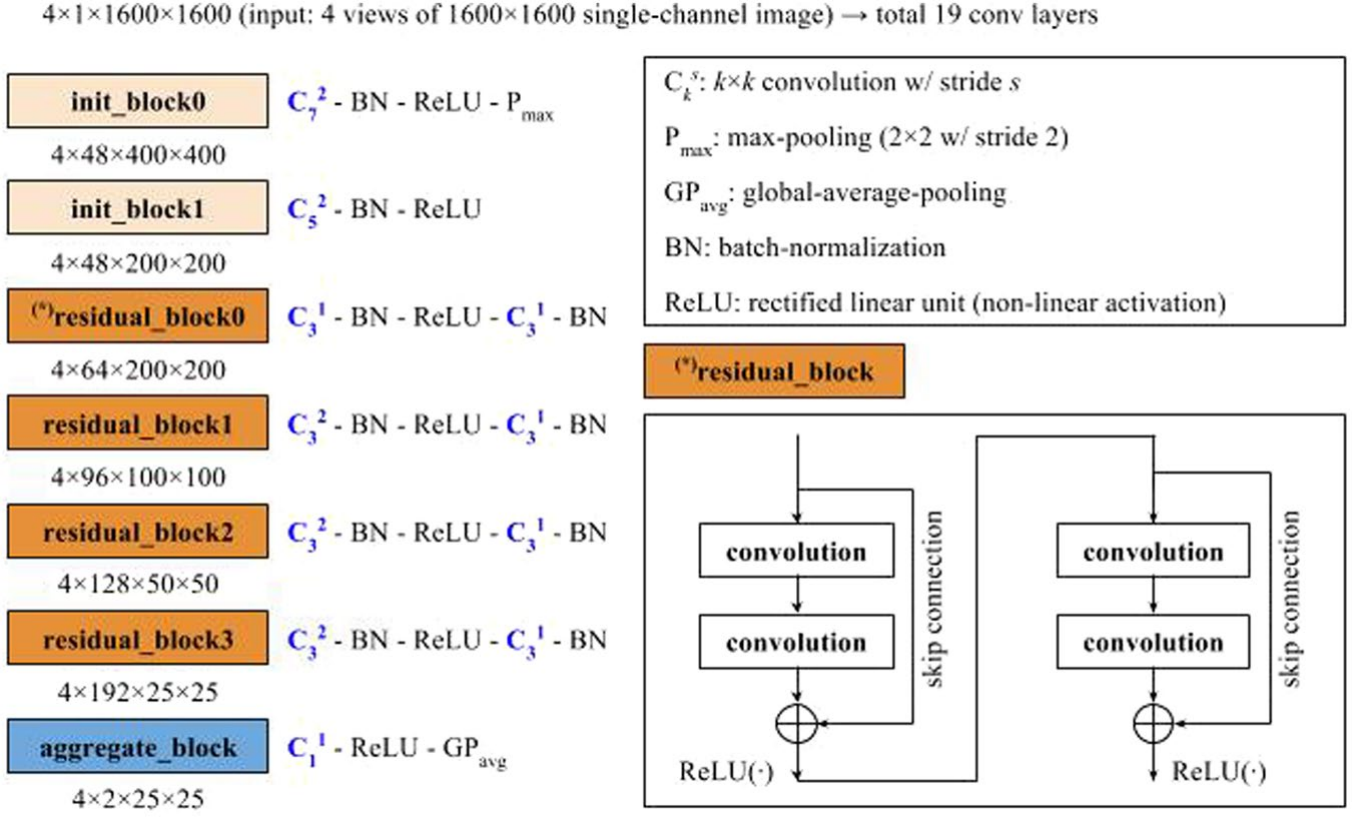
Overall architecture – 19 convolutions followed by a global-average-pooling (GPavg).

Deep convolutional neural network (DCNN) is a deep learning algorithm specialized for images^22^. Each convolutional layer extracts features hierarchically (layer-by-layer) to abstract semantics from the raw input images. DIB-MG is implemented based on a residual network (ResNet)^23^, the state-of-the-art DCNN model for image recognition. Figure 1 shows the overall architecture of DIB-MG. It consists of two initial blocks (init_block), four residual blocks (residual_block), and an aggregation block (aggregate_block). Each residual block includes four consecutive convolution layers with skip connection as described in the right-bottom of the Fig. 1 ($\oplus$ is an element-wise addition operator), while the other blocks include a single convolution layer. Each block also includes some auxiliary components such as a batch normalization layer (BN: normalization of activations within a batch)^24^, a rectified linear unit (ReLU: a simple mathematical function for non-linear activation)^22^, a max-pooling layer (P_max_: static dimension reduction function for translation-invariant feature abstraction)^22^, and a global-average-pooling (GP_avg_: average of the entire 2-dimensional input feature map)^23^. Details of the components are well described in the original literatures^22–24^.

**Figure 2:**
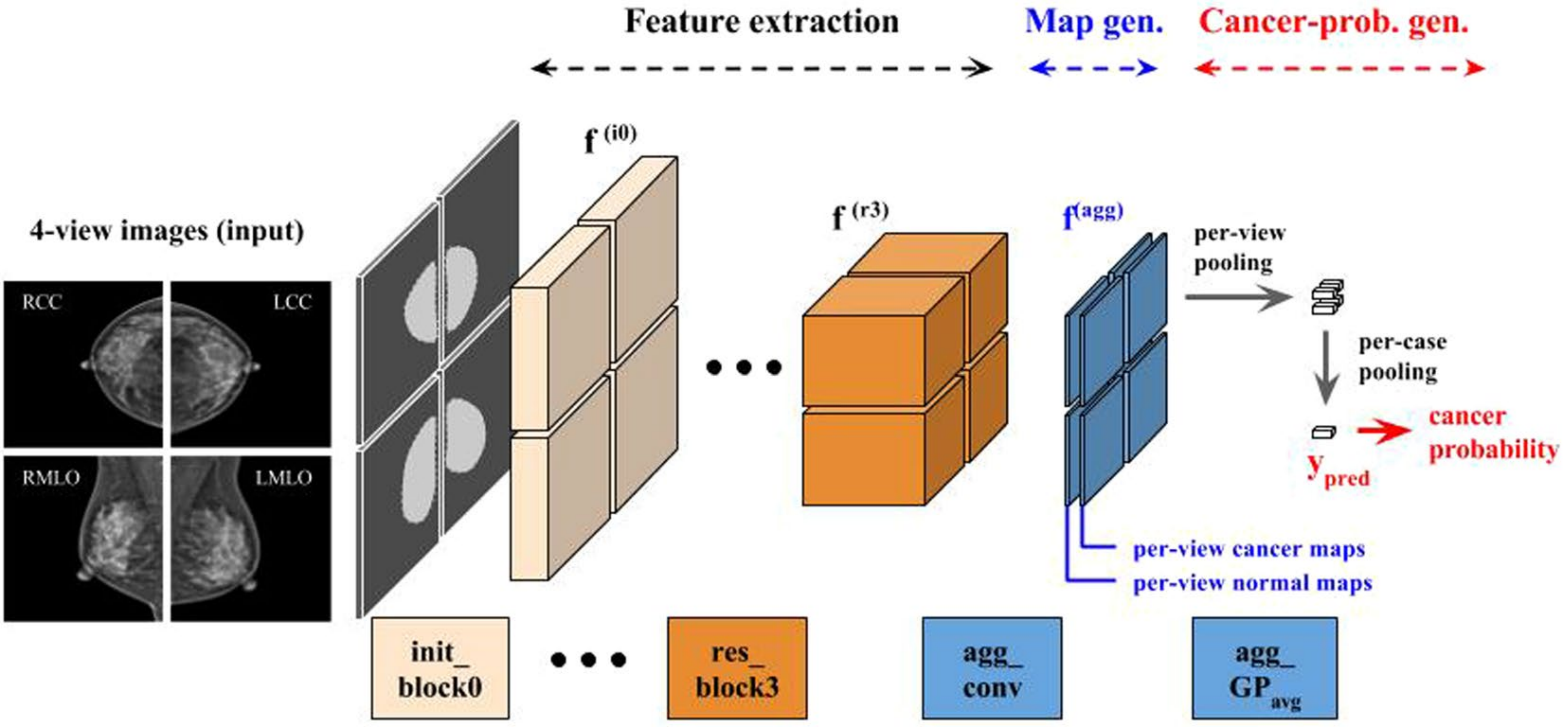
Hierarchical feature abstraction, DIB map generation, and cancer probability generation.

**Figure 3:**
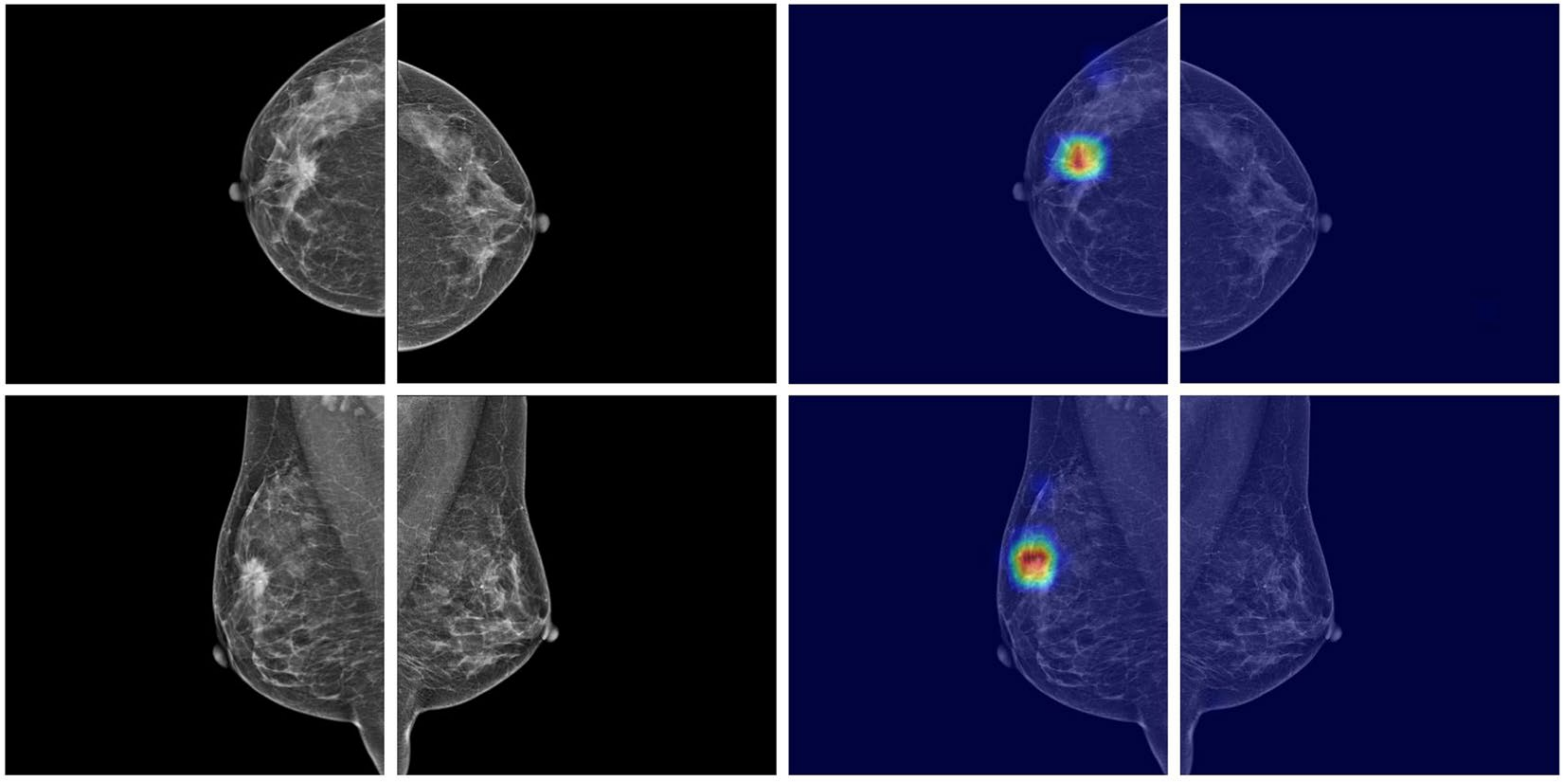
DIB example with ground-truth lesion. A 44-year-old woman with invasive ductal carcinoma of the right breast. A 22 mm-sized mass was correctly highlighted by DIB. The confidence score for cancer of DIB was 1.0 and 0.026 for the right and left breast.

DIB-MG consists of nineteen convolution layers with a two-stage global-average-pooling layer. The former eighteen convolution layers extract hierarchical features for cancer classification, while the last convolution layer (1 × 1 convolution kernel with filter width 2) generates per-view maps (one for cancer, and the other for normal cases) via for final DIB construction (Fig. 2). Figure 3 shows an example of DIB as well as ground-truth lesions. Since we did not use pixel-level lesion annotation in this experiment, each per-view map generated from the last convolution layer (i.e. map generation stage) was converted in a single value to be compared with the ground-truth label (biopsy-proven cancer: 1 or normal: 0). So, the final maps were converted into a vector (each vector element represents its own class) using the global-average-pooling operation. In the training stage, the error between the output vector (y_pred in Fig. 2) and the ground-truth label was propagated backward via back-propagation algorithm^25^, and the model parameters of the entire network were updated based on the propagated errors.

### Training Set-up

All the DICOM files are first converted to PNG files considering window_center and window_width defined in the header of each DICOM, and then the pixel values are normalized to be in the range −1.0 to 1.0. Random perturbation of the pixel intensity in terms of constrast (±10%) and brightness (±10%) is used every training iteration to overcome the difference in vendor-specific contrast/brightness characteristics. All the input images are downscaled to 1600 × 1600, i.e. rescaled to 1600 in terms of the longer side and zero-padded to be 1600 × 1600 (zero-padded on the left side of RCC/RMLO and the right side of LCC/LMLO).

Initial learning rate 0.001 is decayed by a factor of 5 every 10 epochs until the 30 epochs. Stochastic gradient descent (SGD) with momentum 0.9 is used for optimization. Minibatch size is 64 images (16 exams) based on eight graphic processing units (GPUs). Weight decay constant 0.0005 is used for regularization. All the experiment is done with TensorFlow^26^.

### Evaluation of the Algorithm

Training proceeds to minimize the prediction error of the entire training set, and the final DIB-MG performed best on the validation set is chosen for evaluation on the test set. In an inference stage, the final output value of the trained model (y_pred ranging from 0.0 to 1.0) is used to decide whether the input case is cancer or not. More specifically, y_pred represents the confidence level of malignancy. This value is not exactly equal to the probability of cancer, as cancer cannot be specified as a probability. But it is correlated with the cancer probability in real exams. The constructed DIB (class number of per-view maps generated from the last convolution layer) includes information on spatial discriminativity. As mentioned before, each map represents the corresponding class and shows the most discriminative part in terms of the final classification result; e.g., if y_pred is 0.9 (cancer probability), then the region with the highest value on the cancer map is the most discriminative part in terms of its cancer decision.

### Statistical Analysis and Performance Comparison

Chi-square tests were used to see whether there was any difference in categorical variables between training, validation and test sets. With validation and test sets, diagnostic performances were measured. Sensitivity, specificity, and accuracy were compared according to various demographics using the chi-square test. For features, logistic regression with the generalized estimating equation (GEE) method was applied to take into account that some patients had mass with microcalcifications. The AUC were compared between the validation and test sets using chi-square statistics. All analyses were conducted by a statistician using SAS statistical software (version 9.4; SAS Institute Inc., Cary, NC, USA) and R version 3.3.1 (R Foundation for Statistical Computing, Vienna, Austria).

## Results

At the operating point (threshold) of 0.5, sensitivity was 75.6% and 76.1% when specificity was 90.2% and 88.5%, and AUC was 0.903 and 0.906 for the validation and test sets, respectively, with no statistical difference (Table 3). Sensitivity and specificity were not statistically significant between age **≥**50 and **<**50, but they were significantly different according to the manufacturer (Table 3). In regards to breast density, sensitivity was not affected, however, specificity and accuracy decreased as breast density increased (Table 4).

**Table 3:** Diagnostic Performances according to age and manufacturer.

	Sensitivity (%)	Specificity (%)	Accuracy (%)	AUC
Validation Set	75.6 (468/619)	90.2 (558/619)	82.9 (1026/1238)	0.903
Age				
≥50	77.1 (270/350)	91.9 (352/383)	84.9 (622/733)	0.914
<50	73.6 (198/269)	87.3 (206/236)	80.0 (404/505)	0.882
p value^*^	0.310	0.061	0.026	0.080
Manufacturer				
GE	77.5 (186/240)	91.1 (257/282)	84.9 (443/522)	0.924
Hologic	63.6 (126/198)	93.3 (265/284)	81.1 (391/482)	0.863
Siemens	86.2 (156/181)	67.9 (36/53)	82.1 (192/234)	0.861
p value*	**<**0.0001	**<**0.0001	0.2704	
Test Set	76.1 (471/619)	88.5 (548/619)	82.3 (1019/1238)	0.906
Age				
≥50	76.3 (267/350)	90.1 (345/383)	83.5 (612/733)	0.911
<50	75.83 (204/269)	86.01 (203/236)	80.6 (407/505)	0.897
p value*	0.897	0.124	0.189	0.395
Manufacturer				
GE	74.6 (188/252)	89.1 (229/257)	81.9 (417/509)	0.910
Hologic	67.0 (132/197)	92.1 (290/315)	82.4 (422/512)	0.880
Siemens	88.8 (151/170)	61.7 (29/47)	83.0 (180/217)	0.888
p value*	**<**0.0001	**<**0.0001	0.943	

*chi-square test.

**Table 4:** Diagnostic Performances according to breast density.

	Sensitivity (%)	Specificity (%)	Accuracy (%)	AUC
Validation Set	75.6 (468/619)	90.2 (558/619)	82.9 (1026/1238)	0.903
Parenchymal density				
A (n = 59)	81.3 (26/32)	100 (27/27)	89.8 (53/59)	0.946
B (n = 242)	80.9 (110/136)	96.2 (102/106)	87.6 (212/242)	0.950
C (n = 744)	75.0 (234/312)	90.3 (390/432)	83.9 (624/744)	0.900
D (n = 193)	70.5 (98/139)	72.2 (39/54)	71.0 (137/193)	0.790
p value*	0.201	**<**0.001	**<**0.001	
Test Set	76.1 (471/619)	88.5 (548/619)	82.3 (1019/1238)	0.906
Parenchymal density				
A (n = 49)	90.3 (28/31)	100 (18/18)	93.88 (46/49)	0.960
B (n = 252)	75.9 (104/137)	92.17 (106/115)	83.33 (210/252)	0.935
C (n = 744)	75.6 (236/312)	88.43 (382/432)	83.06 (618/744)	0.899
D (n = 193)	74.1 (103/139)	77.78 (42/54)	75.13 (145/193)	0.851
p value^*^	0.301	**<**0.061	**<**0.026	

*Chi-square test.

In the malignant group (Table 5), sensitivity was better in mass than in calcifications (84.1–86.1% vs 77.5–77.9%), better in invasive cancer than in non-invasive cancer (77.0–77.9% vs 54.2–59.7%), and better in mass **≥**20 mm than **<**20 mm (88.5–88.6%, 68.4–71.0%).

**Table 5:** Diagnostic Performances according to malignant characteristics.

	Sensitivity	
Cancer cases	Validation set (n = 619)	Test set (n = 619)
Feature		
mass	84.1 (285/339)	86.1 (292/339)
calcification	77.5 (217/280)	77.9 (218/280)
p value*	0.0385	0.0076
Type		
Invasive	77.9 (422/542)	79.0 (432/547)
Noninvasive	59.7 (46/77)	54.2 (39/72)
p value**	0.0005	**<**0.0001
Size (invasive)		
≥20	88.6 (225/254)	88.5 (222/251)
<20	68.4 (197/288)	71.0 (210/296)
p value**	**<**0.0001	**<**0.0001

*Logistic regression using GEE, **chi-square test.

## Discussions

This is the first study that applies deep learning algorithms in mammography without pixel-level supervision. Our results showed that the AUC values for diagnosing breast cancer using the DIB-MG algorithm were 0.903–0.906, which demonstrates that DIB-MG algorithms can be trained with large-scale data sets without pre-defined mammographic features.

Deep learning algorithm in mammography have been previously studied by several researchers. Wang *et al*. reported that breast cancers presenting microcalcifications could be discriminated by deep learning^27^. They used a previously reported computerized segmentation algorithm in order to extract the clustered microcalcifications from mammograms^28^. In their approach, pre-defined microcalcification features obtained from lesion-annotated mammograms were used as an input for the unsupervised deep learning model (stacked autoencoder)^29^. Kooi *et al*. compared state-of-the art mammography CAD systems, relying on manually designed features as well as data-driven features using DCNN^18^. Especially in a deep learning approach, image patches extracted from lesion-annotated mammograms were used for training. Becker *et al*. evaluated the diagnostic performance of their deep neural network model for breast cancer detection^30^. A total 143 histology-proven cancers and 1,003 normal cases were used for this study, where all the cancer cases of the training dataset were manually annotated pixel-wise by radiologists according to descriptions in the radiology report. Compared to the aforementioned approaches, we used pure data-driven features from raw mammograms without any lesion annotations, which is scalable and practical for future CAD systems.

In previous reports with CAD, sensitivity was higher in microcalcifications than mass^31–34^, however, in this study, sensitivity was better in mass than calcifications. That is due to the difference in data sets. In our data set, both screening and diagnostic mammograms were included, in which 45.7% (1721/3762) of invasive carcinomas were equal or larger than 2 cm, whereas other studies with CAD included only screening mammograms^31–34^. Further studies using the DIB-MG algorithm on screening data sets should follow.

Our data showed that sensitivity for breast cancer detection was similar for non-dense breasts and dense breasts. However, specificity decreased as breast density increased. Eventually, low specificity was directly related with increasing false-positives, so we need to develop algorithms increasing specificity in the future.

In our study, diagnostic performance was different according to the manufacturer; sensitivity is the highest (88.8%) and specificity is the lowest (61.7%) in Siemens. In each data set (training, validation and test sets), the three manufacturers were evenly distributed (roughly 4:3:3 in cancer cases, 5:4:1 in normal cases). However, cancer cases were occupied with 27.2–29.2%, compared to 7.6–8.6% in normal cases in Siemens machine. This indicates that the number of cases trained with a certain type of machine can influence the diagnostic performance of mammography. This kind of selection bias should be considered in a future study regarding deep learning.

We acknowledge several limitations of our study. First, in this study we included only normal and cancer cases, so benign cases need to be included. Also, the dataset should be more expanded. Second, our model does not use any pixel-level annotations for training, so there might be errors in predicting the lesion location in examples predicted as cancer. It is necessary to confirm whether the lesion location is accurately predicted, and retrain the model based on those examples to improve localization performance.

In conclusion, this research showed the potential of DIB-MG as a screening tool for breast cancer. Further studies using a large number of high-quality data including benign cases are needed to further investigate its feasibility as a screening tool.
